# Genetic Variants in Isolated Ebstein Anomaly Implicated in Myocardial Development Pathways

**DOI:** 10.1371/journal.pone.0165174

**Published:** 2016-10-27

**Authors:** Robert J. Sicko, Marilyn L. Browne, Shannon L. Rigler, Charlotte M. Druschel, Gang Liu, Ruzong Fan, Paul A. Romitti, Michele Caggana, Denise M. Kay, Lawrence C. Brody, James L. Mills

**Affiliations:** 1 Division of Genetics, Wadsworth Center, New York State Department of Health, Albany, New York, United States of America; 2 Congenital Malformations Registry, New York State Department of Health, Albany, New York, United States of America; 3 Department of Epidemiology and Biostatistics, University at Albany School of Public Health, Rensselaer, New York, United States of America; 4 Division of Intramural Population Health Research, Eunice Kennedy Shriver National Institute of Child Health and Human Development, National Institutes of Health, Department of Health and Human Services, Bethesda, Maryland, United States of America; 5 Department of Neonatology, Walter Reed National Military Medical Center, Bethesda, Maryland, United States of America; 6 Department of Epidemiology, College of Public Health, The University of Iowa, Iowa City, Iowa, United States of America; 7 Medical Genomics and Metabolic Genetics Branch, National Human Genome Research Institute, National Institutes of Health, Department of Health and Human Services, Bethesda, Maryland, United States of America; National Cancer Center, JAPAN

## Abstract

Ebstein anomaly (EA) is a rare heart defect in which the tricuspid valve is malformed and displaced. The tricuspid valve abnormalities can lead to backflow of blood from the right ventricle to the right atrium, preventing proper circulation of blood to the lungs. Although the etiology of EA is largely unresolved, increased prevalence of EA in those with a family history of congenital heart disease suggests EA has a genetic component. Copy number variants (CNVs) are a major source of genetic variation and have been implicated in a range of congenital heart defect phenotypes. We performed a systematic, genome-wide search for CNVs in 47 isolated EA cases using genotyping microarrays. In addition, we used a custom HaloPlex panel to sequence three known EA genes and 47 candidate EA genes. We identified 35 candidate CNVs in 24 (51%) EA cases. Rare sequence variants in genes associated with cardiomyopathy were identified in 11 (23%) EA cases. Two CNVs near the transcriptional repressor *HEY1*, a member of the NOTCH signaling pathway, were identified in three unrelated cases. All other candidate CNVs were each identified in a single case. At least 11 of 35 candidate CNVs include genes involved in myocardial development or function, including multiple genes in the BMP signaling pathway. We identified enrichment of gene sets involved in histone modification and cardiomyocyte differentiation, supporting the involvement of the developing myocardium in the etiology of EA. Gene set enrichment analysis also identified ribosomal RNA processing, a potentially novel pathway of altered cardiac development in EA. Our results suggest an altered myocardial program may contribute to abnormal tricuspid valve development in EA. Future studies should investigate abnormal differentiation of cardiomyocytes as a potential etiological factor in EA.

## Introduction

Ebstein anomaly (EA) is a rare but serious congenital heart defect (CHD) that was first described in 1866 by Wilhelm Ebstein. EA is characterized by downward displacement of the tricuspid valve resulting from incomplete separation of the tricuspid valve from the underlying myocardium. Right ventricular myocardium abnormalities along with patent foramen ovale, atrial septal defect or right ventricular dilation are present in the majority of EA cases [1]. Although the prevalence of EA was reported in 1958 as 1–5 per 200,000 live births [2], more recent population studies have reported a prevalence of 1 in 13,800–25,600 live births [3–5]. In addition to high intrauterine mortality, 15–50% of neonates born with EA do not survive to age 10 years [1,6].

Although the etiology of EA is largely unresolved, familial recurrence [7] and identification of genetic mutations that segregate with disease provide evidence for a genetic component. Mutations in the sarcomeric protein, myosin heavy chain cardiac muscle beta (*MYH7*), have been found in patients with EA associated with left ventricular non-compaction (LVNC); mutations in *MYH7* show autosomal dominant inheritance with variable penetrance in EA [8–10]. Rare mutations in the cardiac transcription factors NK2 homeobox 5 (*NKX2-5)* and GATA binding protein 4 (*GATA4)* have also been described in EA cases [11–13]. Large chromosomal aberrations and copy number variants (CNVs) have been reported in a small number of EA cases; previously reported variants include deletions at 1p36, 5q35, 8p23.1, 10p, 11q21-23, 18q and duplications of 9p, 11q21-23, 15q, chromosome 18, chromosome 21 and 22q11.2 [3,13–26]. Overall, known genetic causes of EA account for a small proportion of all cases. Here, we describe results from the first genome wide investigation of CNVs in a population-based study of isolated EA cases.

## Methods

### Ethics Statement

The New York State Department of Health Institutional Review Board (NYS DOH IRB) and the National Institutes of Health Office of Human Subjects Research approved this study. Prior to genotyping and analysis, specimens were assigned a random ID number and all personally identifying data were removed. Raw data or genotypes are available upon request with proper IRB approval.

### Case Identification

Cases with isolated EA (no other major birth defects) were identified from among all 2,023,083 resident live births in NYS from 1998 through 2005 reported to the NYS Congenital Malformations Registry (CMR). Reporting of selected major birth defects, including EA, identified during the first two years of life is mandated by NYS law. Methods for case ascertainment by the NYS CMR have been described previously [27]. In a query of the NYS CMR database, 117 EA cases were identified using the expanded British Paediatric Association (BPA) code 746.200 and by searching for the text description “Ebstein”. EA cases known to have syndromes/chromosomal abnormalities (N = 14), other CHDs (N = 37), other major defects (N = 14) or resulting from plural births (N = 1) were excluded from analysis, leaving 51 eligible EA cases. Among these 51 cases, those with common minor CHDs including patent ductus arteriosus (N = 11), patent foramen ovale (N = 5), bicuspid aortic valve (N = 1) or other minor birth defects such as syndactyly of the toes (N = 1) were retained for analysis. We attempted to locate archived newborn screening dried blood spots (DBS) for the 51 eligible EA cases along with three unaffected live births to serve as controls. Sufficient material was available for 47 EA cases, and none had been marked that the parents had refused use of the specimens for research. Demographic data for all NYS live births delivered from 1998 through 2005 were collected from birth records and compared with demographic data for the 47 EA cases using the Pearson Chi-square test in SAS v9.2.

### Genotyping

DNA was extracted from two 3-mm punches of the DBS [28]. A total of 47 EA cases, three unaffected controls, 123 cases with other unrelated phenotypes [29], and one HapMap specimen were genotyped as a single batch. Specimens were genotyped at the Biomedical Genomics Center Core Facility at the University of Minnesota using Illumina HumanOmni2.5–8_v1 bead arrays and the Infinium HD assay protocol. Data were analyzed using Illumina GenomeStudio v2011.1 with a genotype no-call threshold < 0.15.

### Autosomal SNPs

Genotype clusters were defined based on the data generated in this project. Genotypes and clusters were manually reviewed and cleaned by re-clustering, editing, and excluding where appropriate [30]. A total of 2,278,660 autosomal single-nucleotide polymorphisms (SNPs) and 57,201 sex chromosome SNPs passed quality control and were included in CNV analysis (total autosomal SNPs on the array = 2,314,174; total sex chromosome SNPs on the array = 58,187). Among the 2,278,660 autosomal SNPs, the mean specimen call rate among the 47 EA cases and three controls was (± standard deviation, (range)) 99.7% ± 0.3 (98.3%-99.9%) and the mean log R ratio deviation was 0.133 ± 0.030 (0.099–0.231). After cleaning, SNP genotype reproducibility (based on two duplicates included among the 173 samples genotyped) was 100%.

### Sex Chromosome SNPs

SNPs on the X chromosome were clustered using female subjects only and SNPs on the Y chromosome were clustered using male subjects only. SNPs in pseudoautosomal region (PAR) 1 or 2 or in the X-chromosome-transposed region (XTR) were clustered using both males and females. Clusters were reviewed and edited, as described above for autosomal SNPs.

### CNV Calling and Annotation

Autosomal CNVs were called using Illumina's cnvPartition algorithm v3.1.6 and PennCNV v2011/05/03 [31]. For both algorithms, data were GC-wave adjusted, and the minimum number of consecutive probes required for a CNV call was three. The confidence threshold for CNV calling was set to the default value of 35 for cnvPartition and to 10 for PennCNV. For PennCNV calls, the PennCNV function clean_cnv.pl was run with default parameters to merge adjacent CNV calls. Extended regions of copy-neutral homozygosity ≥1 Mb and sex chromosome CNVs were called using cnvPartition. Autosomal CNV call files were annotated using custom C++ programs as previously described [32] to: 1) compare concordance between calling algorithms, 2) count overlapping EA cases and controls, 3) determine overlap with an in-house database of unrelated birth defect case and control CNVs, 4) assess overlap with common HapMap3 copy number polymorphisms [33] and CHOP CNV blocks [34], 5) and identify intersecting transcripts and genes. Transcripts included full-length coding transcripts and full-length non-coding transcripts with a well characterized biotype downloaded from GENCODE (version 19, accessed via UCSC genome browser May 2014) [35]. Genes were defined as those included in the Consensus CDS project (CCDS; release 15, accessed via UCSC genome browser June 2014). CNVs were checked for overlap with previously reported EA genes, *NKX2-5*, *GATA4*, and *MYH7* [10,13]. Sex chromosome CNVs were called with cnvPartition and were manually annotated.

### Candidate Gene Identification

Additional EA candidate genes were identified by searching the disease term ‘Ebstein Anomaly’ in the PhenoDigm database (original query October 2013, same gene list retrieved March 2016), which identifies potential gene-disease associations using phenotype information from a variety of model organisms [36]. Coordinates (hg19) for human orthologs of mouse and zebrafish genes reported by PhenoDigm as associated with EA were obtained using the Ensembl BioMart tool [37] and checked for overlap with all CNVs, as described above.

### CNV Selection and Prioritization

Candidate autosomal CNVs were selected if they met the following criteria: at least 10 probes, at least 25 Kb in length and less than 35% overlap with: common HapMap3 copy number polymorphisms, common CNV blocks identified in the CHOP CNV database, controls in this project (of the same CNV type), other cases with unrelated birth defects and controls that we previously genotyped. The UCSC genome browser [38] was used to filter out CNVs that had substantial overlap with multiple Database of Genomic Variants (DGV) [39] entries (of the same CNV type, release date– 2014/10/16). Sex chromosome CNVs that were at least 25 Kb in length were manually reviewed for overlap with control subjects, other birth defect cases and CNVs catalogued in DGV.

Log-R ratio (LRR) and B-allele frequency (BAF) plots across each candidate CNV region were manually reviewed to subjectively assess the quality and validity of generated CNV calls using the Illumina Genome Viewer in Genome Studio.

### CNV Validation

A subset of the most biologically interesting candidate CNVs were validated in the laboratory using two to three quantitative real-time PCR (qPCR) TaqMan assays (Applied Biosystems, Carlsbad, CA) per region. For detailed information see CNV Validation in S1 Methods.

#### Sequencing

Mutations in 50 genes were screened using a custom Agilent HaloPlex panel. Genes sequenced include: three genes previously associated with EA (*NKX2-5*, *GATA4*, and *MYH7)*, six sarcomere genes, seven genes from candidate EA-associated CNVs identified in this study, 14 highly ranked PhenoDigm [36] genes from regions where one or more EA cases had loss of heterozygosity, nine genes in a critical region for EA identified via linkage study in a canine model [40], six cardiomyopathy genes that are associated with sarcomeric function or LVNC and five genes from a literature review (panel designed using SureDesign, see S6 Table for complete gene list). Sanger sequencing was used to validate potentially pathogenic variants called in HaloPlex data. For detailed information see Sequencing in S1 Methods and S4 Table.

### PhenogramViz CNV Ranking

To validate our CNV selection criteria, PhenogramViz (v0.1.2), a tool for clinical interpretation of CNVs [41], was used to rank the CNVs of all subjects. PhenogramViz utilizes phenotypes and leverages integrated cross-species phenotype ontology to rank the potential pathogenicity of CNVs an individual carries. ‘Ebstein’s anomaly of the tricuspid valve’ was used as the phenotype term. All PennCNV calls that contained at least 10 probes and were at least 25 Kb in length, all cnvPartition autosomal calls at least 25 Kb in length and all cnvPartition sex chromosome calls at least 25 Kb in length that were not copy-number 2 in PAR or XTR regions were ranked using default parameters.

### Gene Set Enrichment Analysis

Three gene lists were generated from GENCODE [35] genes (version 19, accessed via UCSC table browser October 2015) in candidate CNV regions: (1) using all candidate EA-associated CNVs identified in this study; (2) using only candidate EA-associated deletions identified in this study; and (3) using only candidate EA-associated duplications identified in this study. Three known EA genes: *MYH7*, *GATA4* and *NKX2-5* were added to each list. Lists were then separately used as input into Enrichr [42] and results were exported. Tables were filtered to exclude: gene sets with adjusted P ≥ 0.05, gene sets that were enriched with only the three known disease genes, gene sets that were enriched with gene(s) from a single CNV or gene sets that were generated from cell lines not applicable to EA (for example gene sets generated in cancer cell lines). To avoid redundant results, gene sets that were enriched with genes from lists (2) or (3) were excluded if they were also enriched with genes from list (1). After filtering/excluding enriched gene sets, similarities were calculated and visualized with the Enrichment Map Cytoscape app [43] using an overlap coefficient cutoff of 0.5. Connected subclusters were arranged using a degree-sorted-circle layout, scaled to allow label visualization and manually annotated.

## Results and Discussion

### Case Identification

Using BPA code 746.200 and text field descriptions, 117 EA cases were identified in the NYS CMR database from among 2,023,083 NYS live-births, resulting in a birth prevalence of 1 in 17,300. Of the 117 EA cases, 51 were isolated (see methods), resulting in a birth prevalence of 1 in 39,700 live births with isolated EA.

As shown in Table 1, none of the characteristics examined (maternal age, infant sex, maternal race/ethnicity, maternal education, parity, smoking during pregnancy, prenatal maternal body mass index) were statistically different between EA cases and controls.

**Table 1:** Characteristics of isolated EA cases compared to NYS live births.

Characteristic	NYS Live Births (n = 2,023,049)^a^	Ebstein Cases (n = 51)^b^	P Value
**Maternal Age (years)**	**(n = 2,022,740)**	**(n = 51)**	**0.88**
<20	157,085 (7.8)	3 (5.9)	
20–34	1,480,911 (73.2)	38 (74.5)	
≥35	384,744 (19.0)	10 (19.6)	
**Maternal Race/Ethnicity**	**(n = 2,017,837)**	**(n = 51)**	**0.85**
Non-Hispanic White	1,051,561 (52.1)	29 (56.9)	
Black	361,836 (17.9)	8 (15.7)	
Hispanic	437,846 (21.7)	10 (19.6)	
Asian	135,374 (6.7)	4 (7.8)	
Other	31,220 (1.6)	0 (0)	
**Maternal Education (years)**	**(n = 1,997,267)**	**(n = 51)**	**0.91**
<12	384,781 (19.3)	11 (21.6)	
12	594,659 (29.8)	15 (29.4)	
>12	1,017,827 (51.0)	25 (49.0)	
**Parity**	**(n = 2,023,049)**	**(n = 51)**	**0.34**
Nulliparous	846,801 (41.9)	18 (35.3)	
Multiparous	1,176,248 (58.1)	33 (64.7)	
**Maternal Smoking**	**(n = 2,023,049)**	**(n = 51)**	**0.82**
No	1,842,757 (91.1)	46 (90.2)	
Yes	180,292 (8.9)	5 (9.8)	
**Prepregnancy Maternal BMI (kg/m**^**2**^**)**	**(n = 979,285)**	**(n = 25)**	**0.66**
<18.5	40,332 (4.1)	0 (0)	
18.5–24.9	523,438 (53.5)	14 (56.0)	
25–29.9	182,264 (18.6)	6 (24.0)	
≥30	233,251 (23.8)	5 (20.0)	
**Case Sex**	**(n = 2,023,035)**	**(n = 51)**	**0.28**
Male	1,036,825 (51.3)	30 (58.8)	
Female	986,210 (48.8)	21 (41.2)	

Numbers in parentheses represent percentages, unless otherwise indicated. BMI = Body Mass Index.

^a^—Demographic variables were not available for all subjects.

^b^—Includes three isolated Ebstein cases not genotyped.

### CNV Identification

Among the 47 EA cases genotyped, cnvPartition called 1,772 CNVs, and PennCNV called 3,982 CNVs. After applying selection filters, 120 autosomal CNVs and 87 sex-chromosome CNVs were called by one or both algorithms. After manual review and exclusion for substantial overlap with DGV entries, 46 CNV calls in 27 EA cases remained. Upon manual review of LRR and BAF plots, six CNVs in one subject were excluded (duplications/deletions not supported by plot inspection). Two CNVs on the Y chromosome of one male EA case were excluded because they were in genomic regions with sparse representation on the array. After all exclusions, 38 candidate CNVs in 26 EA cases fulfilled our selection criteria. Three of the 38 CNVs were recurrent and 35 were each found in a single EA case. Twenty-six EA cases (55%) carried at least one candidate CNV (Table 2). Seventeen of 20 CNVs selected for validation were confirmed by qPCR, showing the expected copy number for all qPCR assays spanning each region. Two recurrent and one CNV found in a single EA case failed to validate and were considered false positive CNV calls. None of the 17 CNVs that validated were found in 190 control specimens **(**S3 Table**)**.

**Table 2:** Candidate EA-associated CNVs identified in this study.

CNV #	Study ID	Race/Ethnicity	Type (CN,Sex)	Locus	Coordinates (hg19)	~Size (Kb)	Select GENCODE-V19 Transcripts Overlapped*
2^R^^V^	C12	NHB	Dupl	8q21.13	83,573,868–83,964,230	390	*CTD-2272D18*.*1;RP11-653B10*.*1;RP11-731N10*.*1*
2^R^^V^	C47	Asian	Dupl	8q21.13	83,848,688–83,981,829	133	*CTD-2272D18*.*1;RP11-731N10*.*1*
4	C43	NHW	Dupl	1p36.12	21,002,613–21,037,639	35	*KIF17*
5^V^	C4	NHB	Dupl	1p34.1	44,195,344–44,428,902	234	***ARTN****;IPO13;RP11-7O11*.*3;ST3GAL3*
6^V^^L^	C34	NHW	Dupl	1p34.1	46,058,525–46,101,490	43	*CCDC17;GPBP1L1;****NASP***
7	C43	NHW	Dupl	2p23.1	30,656,586–30,735,763	79	***LCLAT1***
8^V^	C22	NHW	Het Del	2q23.1–24.1	149,164,048–156,511,426	7,347	*ARL5A;ARL6IP6;****CACNB4****;****EPC2****;FMNL2;GALNT13;****KCNJ3****;****KIF5C****;LYPD6;LYPD6B;****MBD5****;MIR4773-1;MMADHC;****NEB****;NMI;PRPF40A;RBM43;****RIF1****;RN7SL124P;RNA5SP107;****RND3****;RPRM;SNORD56;STAM2;****TNFAIP6***
9^V^	C20	NHW	Dupl	2q33.1–33.2	203,134,839–203,796,988	662	***BMPR2****;CARF;****FAM117B****;ICA1L;NOP58;RN7SL40P;RN7SL753P;RP11-686O6*.*1;RP11-686O6*.*2;WDR12*
10	C14	NHW	Het Del	3p26.2	3,804,555–3,830,671	26	***SUMF1***
11^V^	C2	HW	Het Del	3p21.1	53,406,740–53,491,422	85	*SNORA26*
12	C23	NHB	Het Del	4q13.1	60,249,365–60,294,193	45	*-*
13^V^	C47	Asian	Dupl	4q26	120,028,773–120,162,705	134	***MYOZ2****;RP11-455G16*.*1;USP53*
14	C10	NHW	Dupl	4q35.1–35.2	187,078,181–187,190,810	113	*CYP4V2;****F11****;FAM149A;****KLKB1***
15	C34	NHW	Dupl	5q22.1	110,417,428–110,444,810	27	*CTC-551A13*.*2;****WDR36***
17	C13	NHW	Het Del	6p25.3	1,831,050–2,175,663	345	***GMDS***
18^V^	C20	NHW	Dupl	6q22.1	118,039,508–118,237,605	198	*SLC35F1*
19	C6	NHW	Dupl	6q24.3	148,005,232–148,138,720	133	*RP11-307P5*.*1;RP11-307P5*.*2*
20^V^	C43	NHW	Het Del	6q25.1	151,427,385–151,655,101	228	***AKAP12****;RN7SKP268;RNU6-1247P;RNU6-300P;RNY4P20;RP1-292B18*.*4;RP1-297M16*.*2*
21^V^	C26	NHB	Het Del	8p23.1	9,258,509–9,298,347	40	*RP11-115J16*.*2*
22^V^	C38	HW	Dupl	8q11.21	48,170,319–48,204,412	34	*SPIDR*
23	C3	NHW	Het Del	8q21.12	79,775,378–80,038,602	263	*AC009941*.*1*
24^V^	C42	NHB	Het Del	9p24.1	7,658,764–7,889,991	231	*RP11-77E14*.*2;TMEM261*
25^V^	C34	NHW	Dupl	10q24.33	105,157,553–105,215,741	58	*CALHM1;CALHM2;PDCD11;RP11-225H22*.*4;RP11-225H22*.*7*
26	C46	HW	Dupl	10q25.1	108,765,792–108,793,878	28	*SORCS1*
27	C34	NHW	Het Del	11p13	35,538,329–35,611,601	73	*PAMR1;RP5-945I17*.*2*
28	C15	Asian	Dupl	11q13.3	69,239,611–69,434,379	195	*AP000439*.*1;AP000439*.*2;AP000439*.*3;AP000439*.*5*
29	C30	HW	Dupl	12p12.1	22,608,226–22,649,460	41	*C2CD5;RP11-359J14*.*2*
30	C35	NHW	Dupl	12q13.12	50,893,815–50,995,674	102	***DIP2B***
31	C4	NHB	Dupl	13q21.32	67,843,475–67,900,578	57	*-*
32^V^	C4	NHB	Dupl	13q22.2	76,247,612–76,281,850	34	***LMO7****;RP11-29G8*.*3*
33^V^	C9	NHW	Dupl	16p13.2	9,041,214–9,295,696	254	*C16orf72;RP11-473I1*.*10;RP11-473I1*.*5;RP11-473I1*.*6;RP11-473I1*.*9;RP11-77H9*.*8;****USP7***
34	C36	NHW	Het Del	16q23.2	80,245,950–80,316,836	71	*RP11-525K10*.*3*
35^V^	C3	NHW	Het Del	19q13.41	52,932,290–52,984,708	52	*ZNF534;ZNF578*
36	C14	NHW	Dupl (3,F)	Xp22.33	902,677–1,262,175	359	*RP11-309M23*.*1*
37^V^	C25	NHW	Dupl (3,F)	Xp11.3	44,381,642–44,872,791	491	*DUSP21;FUNDC1;****KDM6A****;RN7SL291P;RNU6-523P*
38	C42	NHB	Del (0,M)	Xq11.1	61,726,006–62,027,422	301	*-*

Subjects not carrying a candidate EA-associated CNV are excluded from the table. Coordinates shown are from pennCNV calls, except for sex-chromosome CNVs, which are from CNVpartition calls. Abbreviations: NHW—Non-Hispanic, White; NHB—Non-Hispanic, Black; HW—Hispanic, White; CN—Copy number; F—Female; M—Male (only listed for sex-chromosome CNVs).

^L^–One additional subject was found to have extended loss of heterozygosity across candidate region.

^R^–Recurrent CNV (two cases).

^V^–CNV validated by qPCR.

*–Transcripts/genes in bold were listed as potential EA-associated genes in the PhenoDigm database (henceforth, ‘PhenoDigm genes’).

### PhenogramViz CNV Ranking

PhenogramViz was used to rank genes included in all CNVs carried by each individual, based on phenotype, predicted haploinsufficiency and overlap with known benign or pathogenic CNVs. This analysis was carried out post-hoc, and was used to assess our candidate CNV filtering and prioritization methods. Our selection criteria yielded candidate CNVs that were also highly ranked by PhenogramViz (i.e., they included genes likely to be relevant to EA). Of the 38 candidate CNVs identified, 32 were ranked in the top five most likely pathogenic for EA in that individual (S1 Table). Furthermore, 19 of 38 CNVs had a haploinsufficiency score greater than zero, an indication that the CNV overlaps dosage sensitive genes [44]. Overall, the PhenogramViz results suggest that our selection criteria successfully limited our analysis to CNVs predicted to be pathogenic.

### Sequencing Results

Fifty genes, including *NKX2-5*, *GATA4* and *MYH7* which have been previously associated with EA, were sequenced in all 47 EA cases. Filtering criteria differed between the three genes previously associated with EA and 47 candidate EA genes on the panel (details shown in S3 Fig). For the three previously associated genes, filtering resulted in 20 potentially pathogenic variants in 13 EA cases. Of the 20 variants, 13 had an allelic balance <0.25 (heterozygous variants are expected to have a 1:1 ratio of reference to alternate alleles; i.e., an allelic balance of 0.5). The 13 variants with low allelic balance were not expected to validate, and were indeed ruled out by Sanger sequencing. Of the seven variants with expected allelic balance (0.40–0.85), six validated by Sanger sequencing and are shown in Table 3.

**Table 3:** Rare sequence variants validated in known EA genes.

Coordinates (hg19)	Gene	Transcript	Coding DNA Change	AA Change	rsID	ExAC MAF^1^	StudyID
chr5:172,661,963	*NKX2-5*	ENST00000329198	c.124G>C	p.Ala42Pro	rs113818864	1.58E-04	C46
chr14:23,886,518	*MYH7*	ENST00000355349	c.4363G>A	p.Glu1455Lys	N/A	N/A	C37
chr14:23,888,685	*MYH7*	ENST00000355349	c.3853+7C>T	N/A	rs45467397	2.29E-03	C26
chr14:23,900,793	*MYH7*	ENST00000355349	c.732+1G>A	N/A	rs730880850	N/A	C30
chr14:23,900,798	*MYH7*	ENST00000355349	c.728G>A	p.Arg243His	rs267606910	8.24E-06	C19
chr14:23,901,862	*MYH7*	ENST00000355349	c.488A>C^2^	p.Gln163Pro	N/A	N/A	C41

All variants are hetertozygous.

^1^—ExAC v0.3.1 global minor allele frequency.

^2^—SureCall called a homozygous variant, Sanger sequencing revealed a heterozygous variant.

Filtering variants in the remaining 47 genes resulted in 36 potentially pathogenic variants in 18 EA cases. Twelve variants in 11 EA cases were validated by Sanger sequencing and are shown in Table 4. Of the 36 variants tested, 24 did not validate by Sanger sequencing; again, the variants that did not validate had low allelic balance (average 0.27). All variants shown in Tables 3 and 4 are rare or absent from the Exome Aggregation Consortium (ExAC) database, which contains variants from more than: 30,000 European individuals, 8,000 South Asian individuals, 5,000 African American individuals, 5,000 Latino individuals and 4,000 East Asian individuals [45]. In addition, all variants in Table 4 are predicted loss of function, high impact or predicted pathogenic by multiple algorithms (details shown in S3 Fig).

**Table 4:** Rare sequence variants validated in candidate EA genes.

Coordinates (hg19)	*Gene*	Transcript	Coding DNA Change	AA Change	rsID	ExAC MAF^1^	StudyID
chr2:179,446,303	*TTN*	ENST00000589042	c.66692G>A	p.Arg22231His	rs200971254	3.76E-04	C7
chr2:179,575,832	*TTN*	ENST00000589042	c.28131C>A	p.Asn9377Lys	rs72648997	4.23E-05	C4
chr2:179,664,626^2^	*TTN*	ENST00000589042	c.593_595delAAG^2^	p.Glu198del^2^	rs771898264	1.49E-04	C4
chr2:203,420,616	*BMPR2*	ENST00000374580	c.2228A>G	p.Tyr743Cys	rs148257675	3.30E-05	C18
chr4:47,647,166	*CORIN*	ENST00000273857	c.1889G>A	p.Cys630Tyr	rs373155410	8.25E-06	C47
chr12:114,793,401	*TBX5*	ENST00000310346	c.1493C>A	p.Ser498Tyr	N/A	N/A	C45
chr14:23,856,987	*MYH6*	ENST00000356287	c.4505G>A	p.Arg1502Gln	rs199936506	1.81E-04	C14
chr14:23,862,177	*MYH6*	ENST00000356287	c.3195G>C	p.Gln1065His	rs267606904	2.31E-04	C31
chr17:37,821,649^3^	*TCAP*	ENST00000309889	c.37_39delGAG^3^	p.Glu13del^3^	rs397516862	N/A	C2
chr17:39,921,023	*JUP*	ENST00000310706	c.1100G>T	p.Arg367Leu	N/A	N/A	C36
chr19:11,152,089	*SMARCA4*	ENST00000358026	c.4373C>T	p.Thr1458Ile	N/A	N/A	C1
chr19:52,941,827	*ZNF534*	ENST00000332323	c.1153C>G^4^	p.His385Asp	rs201395526	5.48E-04	C11

All variants are heterozygous.

^1^—ExAC v0.3.1 global minor allele frequency.

^2^—SureCall called this variant chr2:179664623:GTACTT>G. Sanger sequencing revealed SureCall’s call is incorrect. The variant in the table was called by Indelligent v.1.2 and then manually annotated.

^3^—SureCall called this variant chr17:37821643:TCGGA>T. Sanger sequencing revealed SureCall’s call is incorrect. The variant in the table was called by Indelligent v.1.2 and then manually annotated.

^4^—SureCall called a homozygous variant, Sanger sequencing revealed a heterozygous variant.

As shown in Table 5, three cases without a candidate CNV carried heterozygous missense *MYH7* mutations that could be pathogenic. Case *C19* carries a *MYH7* variant (p.Arg243His) that is considered pathogenic. This variant was originally reported in an individual with hypertrophic cardiomyopathy (HCM), has subsequently been reported in multiple individuals with left ventricular non-compaction (LVNC) and the arginine 243 position is highly conserved across species (ClinVar RCV000158756.1). Case *C37* carries a variant of uncertain significance (VOUS) in *MYH7* (p.Glu1455Lys). This variant has not been reported in any databases searched and the glutamic acid residue at 1455 is conserved across multiple species, including primates, chicken and zebrafish. Case *C41* carries a *MYH7* VOUS (p.Gln163Pro); this variant is absent from control databases and has been reported in an individual with LVNC [46].

**Table 5:** Candidate EA-associated genetic variants identified in each EA case.

Study ID	Race/Ethnicity	Candidate CNV(s)	Candidate Sequence Variant
C1	NHW	N/A	*SMARCA4—*p.Thr1458Ile
C2	HW	11^V^	*TCAP—*p.Glu13del
C3	NHW	23,35^V^	N/A
C4	NHB	5^V^,31,32^V^	*TTN*—p.Glu198del & *TTN—*p.Asn9377Lys
C6	NHW	19	N/A
C7	NHB	N/A	*TTN*—p.Arg22231His
C9	NHW	33^V^	N/A
C10	NHW	14	N/A
C11	NHW	N/A	ZNF534—p.His385Asp
C12	NHB	2^R^^V^	N/A
C13	NHW	17	N/A
C14	NHW	10,36	*MYH6*—p.Arg1502Gln
C15	Asian	28	N/A
C18	NHW	N/A	*BMPR2—*p.Tyr743Cys
C19	NHB	N/A	*MYH7*—p.Arg243His
C20	NHW	9^V^,18^V^	N/A
C22	NHW	8^V^	N/A
C23	NHB	12	N/A
C25	NHW	37^V^	N/A
C26	NHB	21^V^	*MYH7* - c.3853+7C>T
C30	HW	29	*MYH7* - c.732+1G>A
C31	Asian	N/A	*MYH6*—p.Gln1065His
C34	NHW	6^V^,15,25^V^,27	N/A
C35	NHW	30	N/A
C36	NHW	34	*JUP—*p.Arg367Leu
C37	NHB	N/A	*MYH7*—p.Glu1455Lys
C38	HW	22^V^	N/A
C41	HW	N/A	*MYH7*—p.Gln163Pro
C42	NHB	24^V^,38	N/A
C43	NHW	4,7,20^V^	N/A
C45	NHW	N/A	*TBX5—*p.Ser498Tyr
C46	HW	26	*NKX2-5*—p.Ala42Pro
C47	Asian	2^R^^V^,13^V^	*CORIN*—p.Cys630Tyr

Subjects not carrying a candidate EA-associated CNV or candidate sequence variant are excluded from the table. Abbreviations: NHW—Non-Hispanic, White; NHB—Non-Hispanic, Black; HW—Hispanic, White.

^R^–Recurrent CNV (two cases).

^V^–CNV validated by qPCR.

Three EA cases were found to harbor both a sequence variant in a known EA gene and a candidate CNV (Table 5). EA case *C26* carries a *MYH7* variant in the splice region (c.3853+7C>T) and CNV *#21*. However, considering the low prevalence of EA and the minor allele frequency in ExAC (0.002), it is unlikely that *MYH7* c.3853+7C>T alone causes EA. EA case *C30* also carries a *MYH7* variant (c.732+1G>A) in a canonical splice donor position that has not been reported in the normal population, was reported in an individual with LVNC and in a separate family co-segregated with LVNC in four individuals (ClinVar SCV000054831.2 & SCV000208693.1). It is more likely that the true cause of EA in case *C30* is the *MYH7* splice variant, and that CNV *#29* is a rare CNV not related to EA. Alternatively, both variants may be required for an EA phenotype. Finally, in EA case *C46* we identified a heterozygous missense mutation (p.Ala42Pro) in *NKX2-5*. This variant has previously been reported in an individual with EA, however the variant was also present in the individual’s unaffected father, which was attributed to incomplete penetrance [12]. It is possible *NKX2-5* p.Ala42Pro does not contribute to EA in case *C46*, or that both *NKX2-5* p.Ala42Pro and CNV #26 are required for the EA phenotype.

In addition to the five EA cases with a *MYH7* variant, other cases carry sequence variants in genes associated with autosomal dominant cardiomyopathy. Two EA cases carry missense VOUS in myosin heavy chain cardiac muscle alpha (*MYH6)*. EA case *C14* carries *MYH6* p.Arg1502Gln and EA case C31 carries *MYH6* p.Gln1065His. *MYH6* p.Arg1502Gln has been reported in multiple individuals with dilated cardiomyopathy (DCM), however it was also identified in healthy family members of one proband [47,48]. *MYH6* p.Gln1065His has been reported in an individual with hypertrophic cardiomyopathy (HCM) and was absent from two unaffected family members [48]. EA case *C2* carries a single amino acid deletion (p.Glu13del) in the titin-cap (*TCAP)* gene that has previously been reported in two DCM probands [49]. EA case *C36* carries a VOUS (p.Arg367Leu) in junction plakoglobin (*JUP)* that has not been reported in the normal population databases searched. Dominant mutations in *JUP* have previously been reported to cause arrhythmogenic right ventricular cardiomyopathy [50]. In addition to being associated with cardiomyopathy in humans, both *TCAP* and *JUP* are in a critical region for EA identified via linkage study in a canine model [40]. Finally, three VOUS in titin (*TTN)* were found in two EA cases. Missense variants in *TTN* are challenging to interpret due to a high prevalence of missense variants in the general population and *TTN*s potential role as a DCM modifier gene [51]. However, p.Arg22231His and p.Asn9377Lys are both in the C‐zone of the A‐band in the TTN protein (Uniprot accession: Q8WZ42), a region that has been shown to be enriched for missense variants in DCM cases [51].

Two EA cases have variants in genes that are unique to cushion formation in the atrioventricular (AV) canal (mitral and tricuspid valves), but not in the outflow tracts (OFT) (aortic and pulmonic valves) [52]. EA case *C45* carries a missense T-box 5 (*TBX5)* variant (p.Ser498Tyr). No anomalies other than EA were reported to the NYS Congential Malformations Registry for case *C45*.EA case *C1* carries a missense SWI/SNF related matrix associated actin dependent regulator of chromatin subfamily a member 4 (*SMARCA4*) variant (p.Thr1458Ile). *SMARCA4* regulates cardiac growth and differentiation via cardiomyocyte proliferation [53].

Two sequence variants were detected in genes that were overlapped by CNVs in other EA cases. One case (*C11)* carries a missense zinc finger protein 534 (*ZNF534)* variant (p.His385Asp) and another case (*C18)* carries a missense bone morphogenetic protein receptor type II (*BMPR2)* variant (p.Tyr743Cys).

### CNV Analysis

One EA case (*C26*), carries a heterozygous deletion at 8p23.1 (CNV *#21*; Table 2). Deletions at 8p23.1 have previously been reported in EA [13] and other CHDs [54]. Haploinsuffiency of the cardiac transcription factor *GATA4* is thought to be responsible for CHD seen in individuals with deletions at 8p23.1; however, the CNV identified in EA case *C26* is 2.2 Mb from *GATA4*. Individuals with other CHDs have been reported with 8p23.1 deletions that do not overlap *GATA4* [55]. As previously suggested, altered expression of *GATA4* via positional effects may underlie this association [13,55]. A heterozygous *MYH7* variant (c.3853+7C>T) was also detected in EA case *C26*; however, as discussed, it is unlikely that this variant is pathogenic.

Two EA cases (*C12* and *C47*) had a duplication at 8q21.13 (CNV *#2*) that overlaps two large intergenic non-coding RNAs (lincRNAs). Though not well studied, lincRNAs have been shown, in mice, to coordinate cell-type specific gene expression [56], including activation of a gene regulatory network that includes cardiac transcription factors such as *Nkx2-5*, *Gata4* and *Tbx5* [57]. The CNV *#2* duplications are also 3 Mb upstream of an important cardiac transcription factor *HEY1* [58]. One additional 263 Kb heterozygous deletion at 8q21.12 (CNV *#23* in EA case *C3*) is 650 Kb downstream from *HEY1*. In mice, *Hey* gene expression is required for formation of the boundary between the chamber myocardium and the AV canal myocardium [59]. Embryonic myocardial cells can differentiate into AV canal cardiomyoctes or chamber cardiomyocytes depending on the network of transcription factors expressed in each region. *HEY1* represses transcription by cardiac transcription factors *GATA4* and *GATA6* [58] and represses AV canal gene expression in the chamber myocardium [60]. Although CNVs *#2* and *#23* are distant from *HEY1*, it is possible the CNVs affect expression. For example, significant expression level changes have been observed for genes located up to 6.5 Mb from CNVs that cause Williams-Beuren syndrome, for example [61]. No expression quantitative trait loci (eQTLs) for *HEY1* have been reported in the Genotype-Tissue Expression (GTEx) browser (accessed March 2016) in the regions of the CNV *#2* duplications or the CNV *#23* deletion.

EA case *C47* had a 133 Kb duplication at 4q26 (CNV *#13)* in addition to the 8q duplication. CNV *#13* completely duplicates *MYOZ2*, a sarcomeric protein that is known to cause cardiomyopathy familial hypertrophic type 16 (MIM #613838). In mice, *Myoz2* overexpression protects against cardiac hypertrophy [62]; however, it is unknown whether overexpression is protective in humans or whether the duplication would cause overexpression.

CNV *#33* fully overlaps *C16orf72*, a gene that is highly conserved across species and is not fully duplicated by any CNV catalogued in DGV [39], which is comprised primarily of ‘healthy’ individuals. DECIPHER [63] and ISCA [64] contain CNVs that fully duplicate *C16orf72*; relevant phenotypes from overlapping DECIPHER cases 251553 and 259192 include “Defect in the atrial septum” and “Abnormality of the heart” in ISCA case nssv578696 (S2 Table). Since CNV *#33* is smaller than the CNVs in DECIPHER (254 Kb vs. >11 Mb), our data narrow the critical region for genes effecting cardiac development in this region.

CNV *#37* is a 491 Kb duplication at Xp11.3 that partially overlaps *KDM6A*, a lysine specific demethylase. Haploinsufficiency of *KDM6A* is associated with Kabuki syndrome (MIM #300867). EA has previously been reported in Kabuki syndrome cases [59]. To our knowledge, there have been no reports of KDM6A duplications in Kabuki syndrome. However, depending on the breakpoints, it is possible that the partial duplication causes haploinsufficiency of *KDM6A* by altering the coding frame.

A large 7.3 Mb heterozygous deletion (CNV *#8*) at 2q23.1–24.1 was detected in a single EA case (*C22*). In addition to EA, case *C22* also had syndactyly of toes and bicuspid aortic valve. There has been at least one previous report of an individual with CHD and a 2q23.1 microdeletion [65]. More than 50 transcripts are overlapped by the 2q23.1–24 deletion in EA case *C22*. The highest scoring PhenoDigm gene in the region, *Kcnj3*, causes abnormal myocardial function in mice when knocked out [66]. *TNFAIP6* and *GALNT13*, were also identified as potential EA genes by PhenoDigm. *TNFAIP6*, is a member of the hyaluronan-binding protein family. *TNFAIP6* is expressed in both embryonic and adult cardiac fibroblasts (myocardium) [67], and hyaluronic acid is a major component of the extra cellular matrix in endocardial cushions, the precursors to AV valves [68]. *GALNT13* is a glycosyltransferase enzyme responsible for the O-linked glycosylation of mucins. SNPs in *GALNT13* have been associated with increased tricuspid regurgitation jet velocity [69].

CNVs *#20*, *#24* and *#25* also implicate myocardial development in EA. CNV *#20* is a 227 Kb heterozygous deletion at 6q25.1 that overlaps *AKAP12*. *Akap12* knockout mice show increased cardiac contractility, possibly via increased phosphorylation of *Mybpc3* [70]; in humans *MYBPC3* mutations are known to cause cardiomyopathy (MIM #115197). CNV *#24* is a 231 Kb heterozygous deletion at 9p24.1 that overlaps *TMEM261*. *TMEM261* has been shown to interact with *MEF2C* [71], which is a MADS-box transcription factor that, along with *TBX5* and *GATA4*, plays a role in cardiomyocyte differentiation [72]. Finally, CNV *#25* is a 58 Kb duplication at 10q24.33 ~40 Kb upstream of *NEURL*, which regulates *JAG1* and thus the *NOTCH1* signaling pathway; NOTCH signaling regulates ventricular myocardial development [59].

A novel 662 Kb duplication at 2q33.1–33.2 (CNV *#9*) was detected in a single EA case (*C20)*. CNV *#9* fully duplicates multiple genes, including *BMPR2*. In heart development, *BMPR2* acts as a receptor for *BMP2*, *BMP4* and *BMP7*, and has different spatial roles [52]. Deletion of *Bmpr2* specifically in the endocardium of mice has been shown to result in abnormal tricuspid and mitral valve formation [73]. Furthermore, the gene set ‘*BMPR2*—Kinase Perturbations from GEO’ was significantly enriched for genes overlapped by candidate CNVs (P = 0.01). This finding indicates that a significant number of the genes overlapped by candidate EA CNVs show altered expression in GEO in experiments that perturb *BMPR2* function. As shown in S5 Table, 14 candidate CNVs contain genes whose expression is at least partially controlled by *BMPR2*. Further evidence for the involvement of BMP signaling in the etiology of EA comes from a study showing deletion of *Bmpr1a* in murine cardiac myocytes leads to AV valve defects reminiscent of EA [74]. *BMPR1A* is a type I BMP receptor that forms a complex consisting of two type II and two type I receptors which together, signal downstream to activate SMAD transcriptional regulators. CNV *#11* also implicates BMP/SMAD signaling in EA. CNV *#11* is a small 84.7 Kb heterozygous deletion at 3p21.1 between the *DCP1A* and *CACNA1D* genes. *DCP1A*, is a *SMAD4* interacting protein; *SMAD4* is a downstream target of the BMP signaling pathway involved in cardiac development [75]. In mice, myocardial deletion of *Smad4* has been shown to result in impaired trabeculation and thinning of ventricular myocardium [76].

### Gene Set Enrichment Analysis

We utilized gene set enrichment analysis to discover shared pathways among all genes overlapped by the 36 candidate CNVs in Table 2. In total, 48 gene sets were significantly enriched for genes overlapped by EA CNVs (corrected P < 0.05; S5 Table). Of the 48 enriched gene sets, 10 were enriched for genes overlapped by deletions, nine for genes overlapped by duplications and 29 for genes from a combination of deletions and duplications.

**Fig 1:**
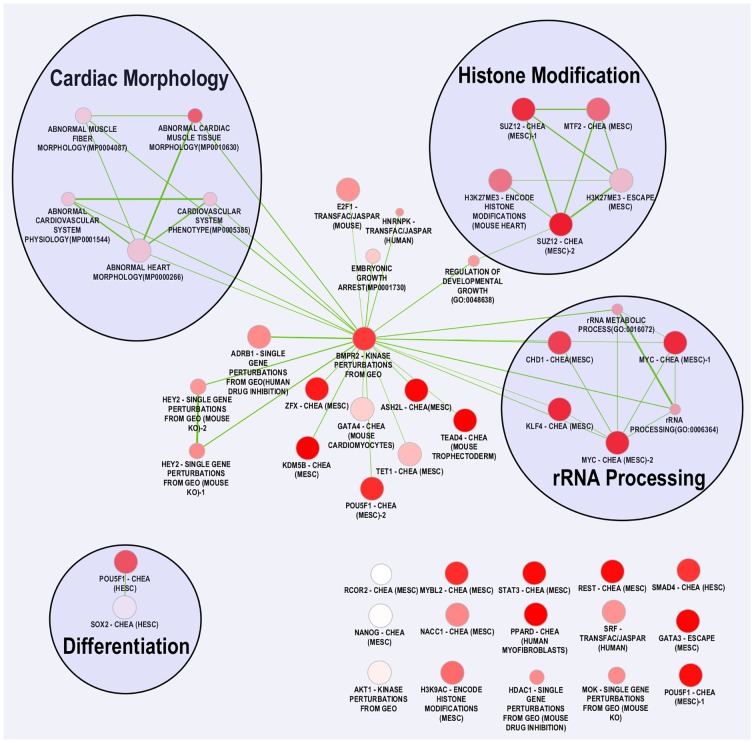
Functional clusters of gene sets enriched with genes in EA CNVs. Network of gene sets that are enriched for genes in candidate EA-associated CNVs. Nodes are Enrichr gene sets that showed significant enrichment of genes in EA-associated CNVs. Clustering was performed with Enrichment Map Cytoscape app to identify enriched gene sets that significantly overlap. Subclusters were manually annotated. Edge weight corresponds to the similarity coefficient between gene sets and node color corresponds to the corrected p-value. Abbreviations: HESC—Human Embryonic Stem Cells, MESC–Mouse Embryonic Stem Cells, CHEA—ChIP Enrichment Analysis, rRNA—Ribosomal RNA, GEO—Gene Expression Omnibus.

One main connected cluster is formed from 31 of the enriched gene sets (Fig 1), each with overlap with the genes in the ‘*BMPR2*—kinase perturbations from GEO’ gene set. In this main cluster, three subclusters of substantially overlapping gene sets are formed. The abundance of connections to the *BMPR2* gene set provides additional support for the involvement of BMP signaling in EA. Furthermore, a ‘cardiac morphology’ subcluster formed by five mammalian phenotype gene sets (Fig 1) supports the involvement of the genes from candidate CNVs in the development of EA. The most statistically significant mammalian phenotype, ‘abnormal cardiac muscle tissue morphology’, specifically supports genetic involvement in the myocardial pathology in EA. Indeed, altered Z-disks in the cardiomyocytes of individuals with EA led Egorova et al. to suggest the presence of Z-disk gene mutations [77].

A ‘histone modification’ subcluster (Fig 1) is formed by two H3K27me3 gene sets, two *SUZ12* gene sets and the *MTF2* gene set. The polycomb repressor complex 2 (PRC2) is a regulator of transcriptional silencing during embryonic development. PRC2 modifies chromatin structure by trimethylating lysine 27 on histone H3 (H3K27me3). *SUZ12* is a component of the PRC2, while *MTF2* recruits PRC2. In mice, overexpression of *Suz12* leads to apoptosis of cardiomyocytes and ventricular non-compaction [78]. The enrichment of gene sets related to histone modification by genes in candidate CNVs suggests histone modification and thus chromatin structure, could play a role in the development of EA. An excess of *de novo* mutations in genes involved in histone modification has been seen in severe CHD cases [79]. Histone modification is also involved in myocardial development and BMP signaling. In the AV canal, *Gata4* together with *Bmp2*/Smad signaling leads to H3K27 acetylation and thus AV canal-specific gene expression. However, in the chamber myocardium, H3K27 deacetylation is necessary to suppress the AV canal-specific gene expression [60].

A ‘rRNA processing’ subcluster (Fig 1), consists of two GO ribosomal RNA (rRNA) processing gene sets, two *MYC* gene sets, the *CHD1* gene set, and the *KLF4* gene set. *MYC* regulates the efficiency of rRNA processing [80] and *CHD1* is required for polymerase I transcription termination [81], which is the polymerase that transcribes rRNA. It is possible these genes alter cardiac protein synthesis via rRNA processing. Although no definitive link between rRNA processing and heart development has been reported, increased *MYC* and rRNA have been shown in human hearts after mechanical unloading [82]. The enrichment of genes related to rRNA processing in candidate CNVs warrants further investigation.

The gene sets ‘*POU5F1*—CHEA (HESC)’ and ‘*SOX2*—CHEA (HESC)’ form a ‘differentiation’ cluster (Fig 1). Both *POU5F1* and *SOX2* have been shown to be involved in cardiac development via differentiation of stem cells to a cardiac cell lineage [83]. In embryonic stem cells, *POU5F1* inhibition prevents differentiation into cardiomyocytes [84]. In addition to the CNV candidate genes identified in the *POU5F1* and *SOX2* gene sets, CNV *#24* has a potential role in cardiomyocyte differentiation (see above).

### Strengths and Limitations

To our knowledge, our study is the first genome-wide study of CNVs in isolated EA cases. Our study is population-based and derived from the large and diverse NYS population (>2 million births in the study period). The NYS CMR has been shown to identify a large percentage of diagnosed cases with reportable birth defects [27]. There are limitations as well. Cases are reported by law, but detailed medical information is not always available in the reports. Moreover, only live births are included and parental DNA was not available to determine if variants identified occurred *de novo*. In addition, our sequence panel did not provide 100% coverage of all targeted genes (S2 Fig); it is possible that regions missed on our panel contain sequence variants relevant to EA. Finally, our study cannot prove pathogenicity of observed CNVs/sequence variants; follow-up studies using animal models or *in vitro* studies will be necessary to confirm that the variants detected in cases contribute to EA.

## Conclusion

The cause of the tricuspid valve abnormality in EA is uncertain. Both failure of the valve to delaminate from the myocardium and abnormalities in myocardial development leading to valve anomalies have been suggested [1,85–87]. Further, it has been suggested that EA may be a cardiomyopathy with valvular involvement rather than a primary valvular disorder [88]. Numerous candidate CNVs identified in this study contain genes linked to myocardial abnormalities or development. Our results suggest that unlike some other birth defects [89], EA is not caused by large recurrent CNVs. However, we found rare, potentially pathogenic CNVs carried by more than one-half of NYS EA cases. Our results support previous findings that genetic factors related to EA are likely heterogeneous. Additionally, we have underscored the potential role of histone-modifying genes in CHD and uncovered rRNA processing as a potential novel pathway involved in the etiology of EA. At least 11 of 35 candidate CNVs identified contain genes related to myocardial abnormalities or development, which may contribute to abnormal tricuspid valve development. This suggests that myocardial development may play an important role in EA. Our results specifically highlight the importance of the BMP pathway in altered myocardial development. Additionally, multiple genes within candidate CNVs and gene set enrichment analysis suggest abnormal differentiation of cardiomyocytes should be investigated as a potential etiological factor. Finally, 11 of 47 EA cases (23%) carry sequence variants in genes associated with autosomal dominant cardiomyopathy; these results further support the role of the myocardium in the pathogenesis of EA.

## Supporting Information

S1 Fig Raw sequencing depth by gene. (PDF)

S2 Fig Percent of bases per gene covered by at least 20x depth. (PDF)

S3 Fig Processing and filtering of HaloPlex variants. (PDF)

S1 Methods Detailed methods for CNV validations, HaloPlex sequencing and Sanger sequencing. (DOCX)

S1 Table PhenogramViz ranking of candidate CNVs. (XLSX)

S2 Table Overlap of candidate CNVs with GWAS loci, DECIPHER and ISCA CNVs. (XLSX)

S3 Table TaqMan copy-number assays used for qPCR validation of candidate CNVs. (XLSX)

S4 Table PCR conditions for Sanger validations of sequence variants. (XLSX)

S5 Table Enrichr results for gene sets enriched for genes in candidate CNVs. (XLSX)

S6 Table Genes targeted by a custom HaloPlex gene panel. (XLSX)
