# Supplementary figures and images for: Genetic Variants in Isolated Ebstein Anomaly Implicated in Myocardial Development Pathways
Source: PLoS One. 2016 Oct 27;11(10):e0165174. doi: 10.1371/journal.pone.0165174 (PMC5082909; doi:10.1371/journal.pone.0165174)

S1 Fig - Raw sequencing depth by gene

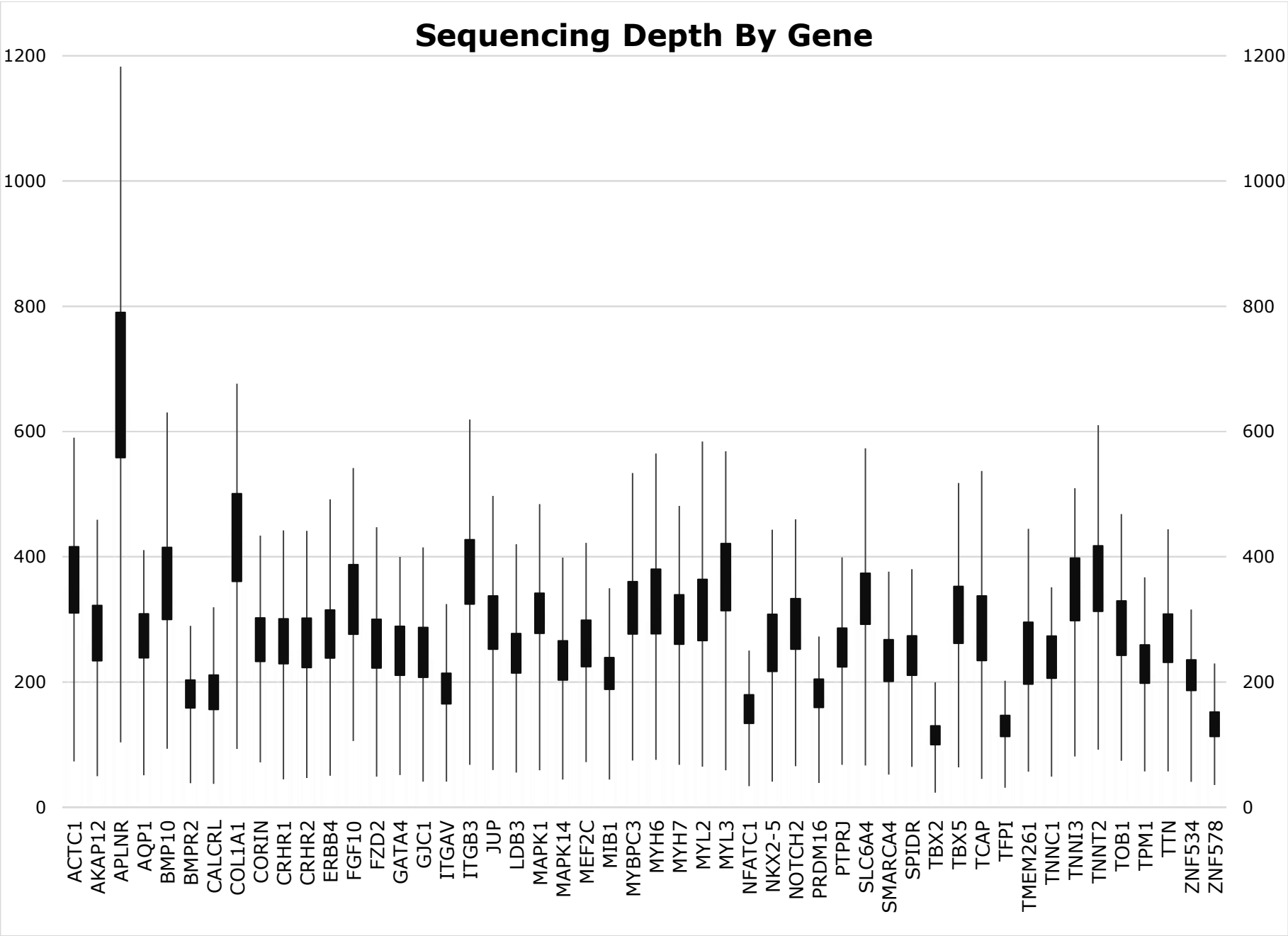

Supplement: S1 Fig — (PDF) [file pone.0165174.s001.pdf]

S2 Fig - Percent of bases per gene covered by at least 20x depth

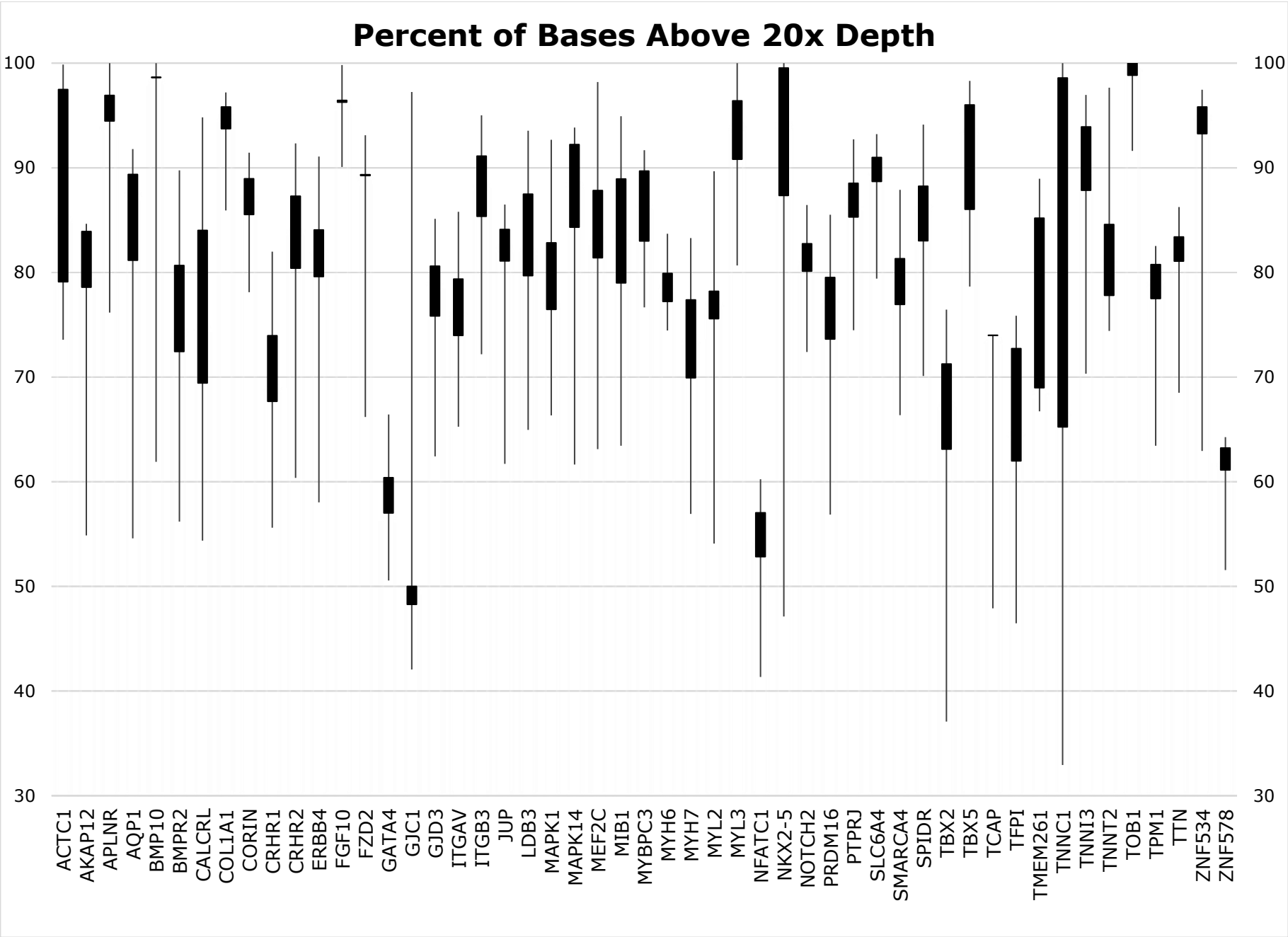

Supplement: S2 Fig — (PDF) [file pone.0165174.s002.pdf]
