# Supplementary material for: Genetic Variants in Isolated Ebstein Anomaly Implicated in Myocardial Development Pathways
Source: PLoS One. 2016 Oct 27;11(10):e0165174. doi: 10.1371/journal.pone.0165174 (PMC5082909; doi:10.1371/journal.pone.0165174)
Supplement: S3 Fig — (PDF) [file pone.0165174.s003.pdf]

S3 Fig - Processing & filtering of HaloPlex variants

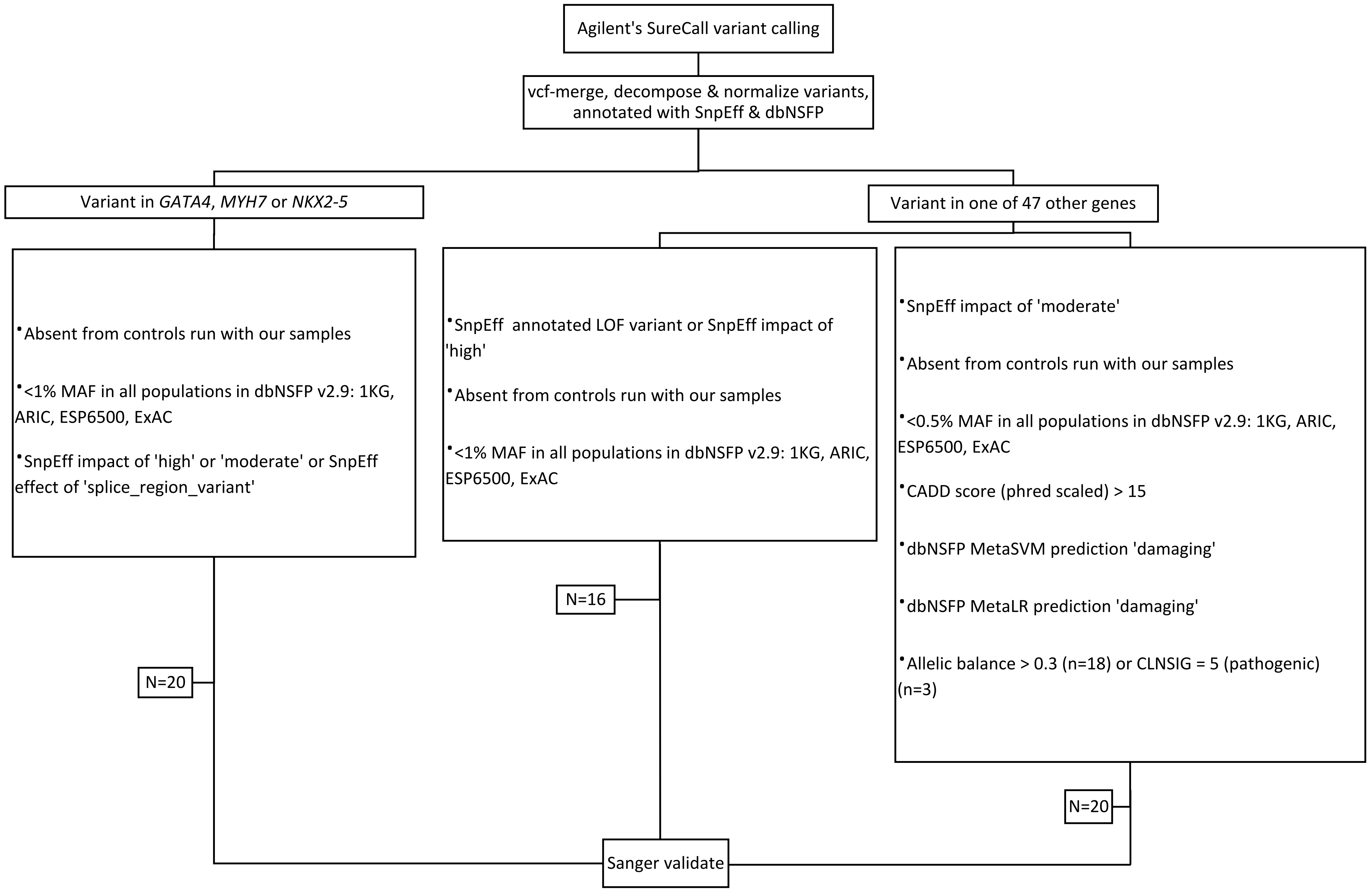


Variants were required to fulfill all requirements in each box. Abbreviations: MAF = Minor allele frequency; dbNSFP = database for nonsynonymous SNPs' functional predictions; 1KG = 1000 genomes phase 1; ARIC = Atherosclerosis Risk in Communities Study; ESP6500 = National Heart, Lung, and Blood Institute Grand Opportunity Exome Sequencing Project; ExAC = Exome Aggregation Consortium; CADD = Combined Annotation Dependent Depletion; MetaSVM = Meta Support Vector Machine (ensemble prediction score from dbNSFP); MetaLR = Meta logistic regression (ensemble prediction score from dbNSFP); CLNSIG = Clinical Significance score in ClinVar.
