# Supplementary material for: Genetic Variants in Isolated Ebstein Anomaly Implicated in Myocardial Development Pathways
Source: PLoS One. 2016 Oct 27;11(10):e0165174. doi: 10.1371/journal.pone.0165174 (PMC5082909; doi:10.1371/journal.pone.0165174)
Supplement: S1 Methods — (DOCX) [file pone.0165174.s004.docx]

***CNV Validation***

Genomic DNA was extracted from one 3-mm DBS [10], diluted 1:10 in water, and amplified using TaqMan Environmental Master Mix (ABI) in 5 µl reaction volumes. A fragment of the RNaseP H1 RNA gene was co-amplified and used as an internal control (TaqMan Copy Number Reference Assay, ABI). Assays were run in quadruplicate on either an ABI 7900HT or an ABI QuantStudio. CopyCaller software v2.0 (ABI) was used to analyze the real-time data using relative quantitation (2-ΔΔCt method). The manual Ct threshold was set to 0.2 with the automatic baseline on. CopyCaller software parameters were as follows: the median ΔCt for each experiment was used as the calibrator, wells with an RNaseP Ct > 38 were excluded and the zero copy ΔCt threshold was set to 6. The average copy number and a software-generated confidence value were calculated for each subject. Samples with confidence values ≥ 0.95 were considered valid; samples with confidence values <0.95 were rerun in quadruplicate. Multiple assays targeted each CNV, and in all cases, no single sample contained all low confidence calls throughout a CNV region. One probe (Hs05075633_cn) was excluded due to discordant results obtained when retesting multiple samples with low confidence calls (Table 3 in Supporting Information S1). All assays were tested in each of the 47 cases and in 32 control subjects. We subsequently screened all validated CNVs against an additional 158 race/ethnicity matched control samples from unaffected NYS births using at least one assay targeting the area of interest. Therefore, a total of 190 unaffected controls were screened using at least one assay in the candidate CNV region.

***Sequencing***

HaloPlex libraries were generated according to manufacturer protocol, except that ~80 ng of DNA per sample was used and 2 extra polymerase chain reaction (PCR) cycles were added. Following library preparation 26, 24 and 29 samples were multiplexed in three separate batches and run on a 300 cycle v2 MiSeq flow cell (the final group of 29 samples included 26 samples of a different cohort). Raw sequencing data was demultiplexed and adapters trimmed using CASAVA (v1.8.2) to generate forward and reverse read FASTQ files for each sample. FASTQ files were processed using Agilent’s SureCall software (v3.0.1.4) using the default HaloPlex parameters; SureCall uses BWA-MEM (v3.0.0) for alignment and SNPPET for variant calling. The average number (± standard deviation, range) mapped reads per individual was 1,359,373 (± 325,982, (276,297- 2,253,325)). The average number of reads in the sequenceable region was 1,339,324 (± 318,763, (271,688- 2,199,223)). Mean read depth was 464X (± 111X, (92X-758X)). 95.6% (± 1.8%, (87.4%-97.1%)) of bases had ≥20X and 73.4% (± 11.4%, (8.1%-85.4%)) of bases had ≥200X coverage (S8 & S9 Figures). VCF files for each sample were merged using the vcf-merge function in vcftools (v0.1.12b). Next, variant normalization was performed using the decompose followed by the normalize functions of vt (v0.57). Normalized variants were annotated with SnpEff (v4.1g) and included annotations from the database for nonsynonymous SNPs' functional predictions (dbNSFP v2.9) [41,42]. Annotated variants were filtered to identify potentially pathogenic variants using SnpEff. *GATA4*, *MYH7* or *NKX2-5* variants were deemed potentially pathogenic if they fulfilled the following requirements: absent from four controls run with our samples; less than 1% minor allele frequency in all populations in dbNSFP v2.9 (1000 genomes phase 1, Atherosclerosis Risk in Communities Study (ARIC), NHLBI GO Exome Sequencing Project (ESP6500 data set) and Exome Aggregation Consortium (ExAC)); SnpEff impact of ‘high’ or ‘moderate’ or SnpEff effect of ‘splice_region_variant’ (S10 Figure). Following SnpEff filtering, the remaining variants were manually reviewed to determine potentially pathogenicity. Variants in genes other than *GATA4*, *MYH7* or *NKX2-5* were deemed potentially pathogenic if they fulfilled all of the following sets of requirements: absent from four controls run with our samples; less than 1% minor allele frequency in all populations in dbNSFP v2.9 (1000 genomes phase 1, Atherosclerosis Risk in Communities Study (ARIC), NHLBI GO Exome Sequencing Project (ESP6500 data set) and Exome Aggregation Consortium (ExAC)); SnpEff impact of ‘high’ or annotated loss of function (LOF) or absent from four controls run with our samples; less than 0.5% minor allele frequency in all populations in dbNSFP v2.9 (1000 genomes phase 1, Atherosclerosis Risk in Communities Study (ARIC), NHLBI GO Exome Sequencing Project (ESP6500 data set) and Exome Aggregation Consortium (ExAC)); SnpEff impact of ‘moderate’; Combined Annotation Dependent Depletion (CADD) score (phred scaled) > 15; dbNSFP MetaSVM prediction is ‘damaging’; dbNSFP MetaLR prediction is ‘damaging’; alternate allele frequency > 0.3 or clinvar pathogenic (S10 Figure).

To validate potentially pathogenic variants called in HaloPlex data, Sanger sequencing was performed. PCR reactions contained extracted DNA, DNA Master HybProbe master mix (Roche Applied Science; Indianapolis, IN), 2.5 mM MgCl_2_, and 0.2 µM each primer, in a total volume of 25 µl or extracted DNA, AmpliTaq Gold 360 Master Mix (Applied Biosystems; ABI; Carlsbad, CA), and 0.2 µM each primer, in a total volume of 25 µl (S4 Table). Standard cycling conditions for DNA Master HybProbe master mix included an initial denaturation at 95°C for 5 minutes, 35 cycles of denaturation at 95°C for 30 seconds, annealing at the specified annealing temperature (S4 Table) for 30 seconds, elongation at 72°C for 30 seconds, and a final extension at 72°C for 5 minutes. Standard cycling conditions for AmpliTaq Gold 360 Master Mix included an initial denaturation at 95°C for 10 minutes, 35 cycles of denaturation at 95°C for 30 seconds, annealing at the specified annealing temperature (S4 Table) for 30 seconds, elongation at 72°C for 60 seconds, and a final extension at 72°C for 7 minutes. PCR products were cleaned-up using ExoSAP-IT (USB Corporation; Cleveland, Ohio), and sequenced using BigDye Terminator v.3.1 Cycle Sequencing chemistry kits (ABI) on an ABI 3730 DNA Analyzer. Sequence chromatograms were analyzed using SeqScape v.2.1.1 (ABI), FinchTV v.1.4.0 (Geospiza; Seattle, WA), BLAT on the UCSC genome browser and Indelligent v.1.2 [1], when necessary.

1. Dmitriev DA, Rakitov RA. Decoding of superimposed traces produced by direct sequencing of heterozygous indels. PLoS Comput Biol. 2008 Jul 25;4(7):e1000113.
